# In Vitro Cell Model Investigation of Alpha-Synuclein Aggregate Morphology Using Spectroscopic Imaging

**DOI:** 10.3390/ijms252212458

**Published:** 2024-11-20

**Authors:** Priyanka Swaminathan, Therése Klingstedt, Vasileios Theologidis, Hjalte Gram, Johan Larsson, Lars Hagen, Nina B. Liabakk, Odrun A. Gederaas, Per Hammarström, K. Peter R. Nilsson, Nathalie Van Den Berge, Mikael Lindgren

**Affiliations:** 1 Department of Physics, Faculty of Natural Sciences, Norwegian University of Science and Technology (NTNU), Gløshaugen, Realfagbygget, NO-7491 Trondheim, Norway; priyanka.swaminathan@ntnu.no; 2 Department of Physics, Chemistry and Biology, Linköping University, SE-581 83 Linköping, Sweden; 3 Department of Clinical Medicine—Core Center for Molecular Morphology, Section for Stereology and Microscopy, Aarhus University, 8000 Aarhus, Denmark; 4 DANDRITE, Danish Research Institute of Translational Neuroscience & Department of Biomedicine, Aarhus University, 8000 Aarhus, Denmark; 5 PROMEC—Proteomics and Modomics Experimental Core Facility at NTNU and the Central Norway Regional Health Authority, NO-7491 Trondheim, Norway; 6 Department of Clinical and Molecular Medicine, NTNU Norwegian University of Science and Technology, NO-7491 Trondheim, Norway; 7 Division of Natural Sciences, University of Agder, NO-4630 Kristiansand, Norway

**Keywords:** αsyn aggregate morphologies, HEK293 cells, FLIM, LCO, h-FTAA

## Abstract

Recently, it has been hypothesized that alpha-synuclein protein strain morphology may be associated with clinical subtypes of alpha-synucleinopathies, like Parkinson’s disease and multiple system atrophy. However, direct evidence is lacking due to the caveat of conformation-specific characterization of protein strain morphology. Here we present a new cell model based in vitro method to explore various alpha-synuclein (αsyn) aggregate morphotypes. We performed a spectroscopic investigation of the HEK293 cell model, transfected with human wildtype-αsyn and A53T-αsyn variants, using the amyloid fibril-specific heptameric luminescent oligomeric thiophene h-FTAA. The spectral profile of h-FTAA binding to aggregates displayed a blue-shifted spectrum with a fluorescence decay time longer than in PBS, suggesting a hydrophobic binding site. In vitro spectroscopic binding characterization of h-FTAA with αsyn pre-formed fibrils suggested a binding dissociation constant K_d_ < 100 nM. The cells expressing the A53T-αsyn and human wildtype-αsyn were exposed to recombinant pre-formed fibrils of human αsyn. The ensuing intracellular aggregates were stained with h-FTAA followed by an evaluation of the spectral features and fluorescence lifetime of intracellular αsyn/h-FTAA, in order to characterize aggregate morphotypes. This study exemplifies the use of cell culture together with conformation-specific ligands to characterize strain morphology by investigating the spectral profiles and fluorescence lifetime of h-FTAA, based upon its binding to a certain αsyn aggregate. This study paves the way for toxicity studies of different αsyn strains in vitro and in vivo. Accurate differentiation of specific alpha-synucleinopathies is still limited to advanced disease stages. However, early subtype-specific diagnosis is of the utmost importance for prognosis and treatment response. The potential association of αsyn aggregates morphotypes detected in biopsies or fluids to disease phenotypes would allow for subtype-specific diagnosis in subclinical disease stage and potentially reveal new subtype-specific treatment targets. Notably, the method may be applied to the entire spectrum of neurodegenerative diseases by using a combination of conformation-specific ligands in a physicochemical environment together with other types of polymorphic amyloid variants and assess the conformation-specific features of various protein pathologies.

## 1. Introduction

Parkinson’s disease (PD) and other related synucleinopathies are characterized by pathological misfolding and accumulation of the αsyn protein into insoluble aggregates. The disease-associated αsyn protein aggregates are known to form distinct structural morphotypes attributing to clinical heterogeneity and pathological complexities in the various synucleinopathies [1,2,3]. Moreover, it has been hypothesized that morphological differences may stem, at least in part, from the intrinsic cellular milieu in which the protein aggregation occurs [2,4,5]. However, the exact link between aggregate morphology and cellular environments remains to be elucidated.

Although mounting evidence points towards the existence of various polymorphic forms of αsyn assemblies [6,7,8,9,10,11], the detection and differentiation of these in varying cellular environments remains challenging using conventional methods of protein aggregate characterization [12,13]. Thiophene-based fluorescent ligands, also known as luminescent conjugated oligothiophenes (LCOs), have been extensively characterized for detecting various polymorphic variants of protein aggregates [14,15,16,17]. The conformational flexibility of the thiophene backbone of these LCOs becomes constricted when they bind to the repetitive cross β-sheet structure of the protein aggregates [14]. This impacts their spectral properties, which can change based on variations in the conformation of the aggregates, making the LCOs suitable for assessing αsyn aggregate morphologies based on their spectral features in a cellular environment under in vitro conditions. For instance, LCOs have been reported to differentiate between αsyn protein aggregates in brain tissue sections of PD and multiple system atrophy (MSA) patients [17]. Furthermore, to evaluate the structural features of protein aggregates in varying chemical environments, such as within cells, it is essential to understand the surrounding solvent environment, which can be achieved by assessing fluorescence lifetime [18,19,20]. In addition to displaying distinct spectral signatures, LCOs also exhibit unique fluorescence lifetimes characteristic of the protein deposits to which they bind [17,21]. Here, we aim to assess spectral profiles, as well as the lifetime signatures of an LCO, when binding to αsyn aggregates in an in vitro environment involving cell culture to better understand whether any changes in the spectral properties and the associated lifetime distributions are influenced by inherent cellular environment. Such knowledge is crucial to understanding clinical heterogeneity in synucleinopathies, where the cellular environment of the first aggregate may contribute to aggregate morphology and clinical phenotype [22].

In this study, the heptameric LCO, heptamer formyl thiophene acetic acid (h-FTAA) [14], was used. This ligand displays distinctive lifetime distributions when it binds to αsyn aggregates in brain tissue sections of PD and MSA patients [17]. Moreover, differences in the decay times of lifetime profiles from h-FTAA have been reported upon binding to pathogenic prions in mouse brain tissue sections and amyloid-β deposits in transgenic mouse models [14,21].

Given the versatility of the LCOs to detect and distinguish morphologies of αsyn aggregates, here we aimed to explore this approach of utilizing h-FTAA together with recombinant αsyn pre-formed fibrils (PFFs) to assess the ligand’s binding ability in a physiologically controlled environment. Taking this approach further, we used human embryonic kidney (HEK293) cells, which were exogenously introduced with either human-A53T-mutated-αsyn (A53T) [23,24,25,26] or human wildtype-αsyn (WT), to induce αsyn aggregation upon exposure to PFFs. This allowed us to examine the spectral profiles and lifetime distributions of h-FTAA bound to αsyn aggregates in an in vitro cellular environment, aiming to get insights into the role of the inherent cellular environment upon the conformation of aggregates and the photophysical response of fluorescent ligands.

## 2. Results

### 2.1. Characterization of Photophysical Properties of h-FTAA in Different Solvent Environments

The synthesis and the excitation and emission profiles of h-FTAA in PBS have been previously reported, with h-FTAA showing excitation at 480 nm and emission at around 548 nm [27]. In order to elucidate the binding/behavior of h-FTAA to PFFs, we also examined the general photophysical properties of h-FTAA in the protic solvents methanol and ethanol, in addition to PBS. This was to mimic a solvation structure of less polarity surrounding the fluorescent ligand, since this is generally the case for amyloid binding sites. The absorption spectra of h-FTAA show similar bands with maxima in the 420–440 nm range for all three polar solvents (Figure S1A). The emission profiles of h-FTAA in PBS and methanol show emission maxima around 550 nm, while in the more hydrophobic ethanol the vibrational substructure is more resolved along with a broad, red-shifted shoulder towards 580 nm, which is more similar to the spectrum of h-FTAA when bound to amyloid (see e.g., [27]). The fluorescence decay time of h-FTAA in the solvents was measured using time-correlated single photon counting (TCSPC), with the sample being excited by a 455 nm LED and the fluorescence monitored at the emission maxima, as outlined above. The decay traces (Figure S1B) all showed contributions from at least two components, indicating that the heptameric h-FTAA probably exists in several differently twisted conformations in the protic solvents. The decays were analyzed, assuming two time components (*τ_i_*), each contributing with relative amplitudes (*B_i_*), as shown in Table 1. The intensity of average decay time was found to be 440, 680, and 740 ps for h-FTAA in PBS, methanol and ethanol, respectively. Furthermore, quantum yield (QY) for h-FTAA was determined in these polar solvents using Coumarin153 as a reference [28]. The slope plots for evaluating QY for h-FTAA in different solvents are shown in Figure S1C, D. The QY was calculated to be over 40% in ethanol while in methanol and PBS, it was considerably lower, ranging from 6% to 11% (Table 1). It is noted that the lifetimes shorten concomitantly with the lowering of the QY due to aqueous solvent quenching, as has been observed for related fluorescent amyloid ligands [29].

### 2.2. In Vitro Spectroscopic Evaluations of h-FTAA Binding to Human-αsyn Pre-Formed Fibrils

We first verified that the pre-formed αsyn fibrils (PFFs), frozen at −80 °C and thawed, showed the expected amyloid fibril morphology by negative stain transmission electron microscopy (TEM). The overall fibril structure appeared intact (Figure S2). As the PFF samples were sonicated before freezing, the fibrils were predominantly composed of fragmented fibrils (<500 nm in length) with some lateral association between fibrils.

**Figure 1:**
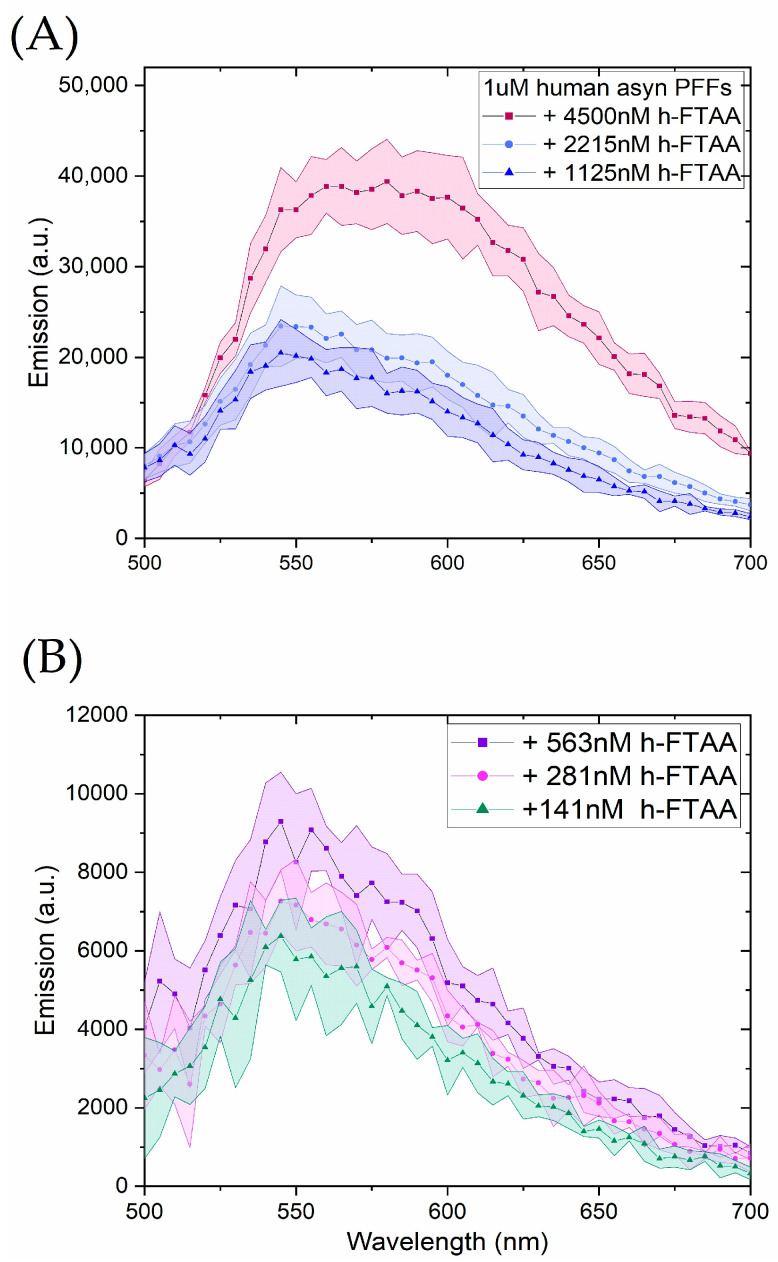
Emission spectra of h-FTAA binding to PFFs fixed at 1 µM concentration with varying concentrations of h-FTAA from (**A**) 1125 nM to 4500 nM to (**B**) 141 nM to 563 nM. The samples were excited at 450 nm and the emission was recorded in the range of 500–700 nm. Each spectrum was baseline corrected using h-FTAA emission in PBS only, respectively. The shaded region in each spectrum represents the standard deviation from triplicates of the varied concentrations of h-FTAA while keeping the concentration of PFFs fixed at 1 µM.

To investigate the binding efficiency of h-FTAA with recombinant human αsyn PFFs, so-called binding curves were measured by varying the concentration of h-FTAA (0–4500 nM) while keeping the concentration of PFF fixed at 1 µM (on the αsyn monomer basis). The corresponding spectral profiles of h-FTAA upon binding to αsyn PFFs are presented in Figure 1. For better data representation, the spectral profiles of h-FTAA are illustrated in two separate plots: with Figure 1A showing high h-FTAA loading (1125–4500 nM), and Figure 1B depicting low h-FTAA loading (141–563 nM). The shaded regions represent the standard deviation from triplicates of h-FTAA concentration loading, while keeping the protein concentration constant. The spectra all show a characteristic emission maximum in the 545–570 nm range with a notable broad shoulder towards the red spectral region growing in at the higher h-FTAA loadings (Figure 1A,B). Upon exciting the sample at 450 nm, the emission intensity of h-FTAA bound to PFFs increased progressively with increasing concentrations of h-FTAA, to be elaborated more below. The excitation profiles were also assessed, provided in Figure S3, showing broad feature-less bands in agreement with the absorbance spectra of 450–470 nm (Figure S1A). Further results on αsyn amyloid fibrils in the cell model are outlined in Section 2.3 and Section 2.4.

**Figure 2:**
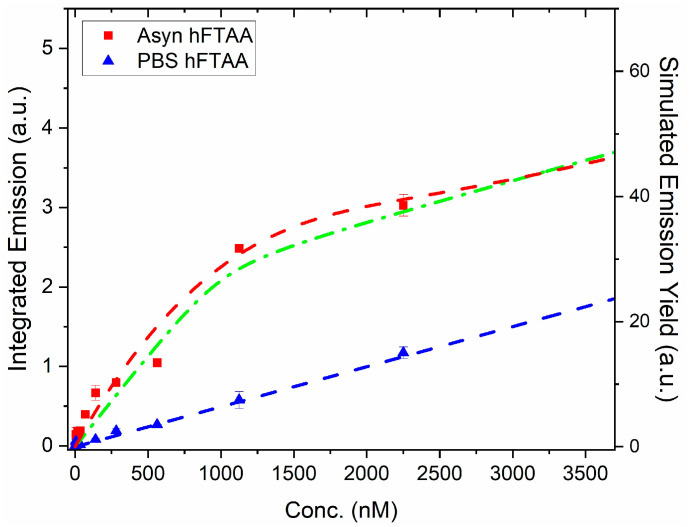
Binding curve of PFFs (1 μM) vs. h-FTAA concentration (red squares). The blue triangles show the signal obtained from only h-FTAA in PBS. The excitation wavelength was 450 nm, and the spectra were collected as in Figure 1. The dashed curves are simulations where the blue dashed line corresponds to 6% QY of h-FTAA in PBS (Table 1). Green dot-dashed: 1-site binding K_d_ = 25 nM; QY 30%. Red dashed: two-site model, K_d1_ = 100 nM; K_d2_ = 300 nM. QY(h-FTAA/PFF-site1) 40%; QY(h-FTAA/PFF-site2) 20%. For details of the two-site model, see [32].

Furthermore, to assess the binding of h-FTAA to the PFFs, the emission spectra were integrated and plotted against the concentration of h-FTAA, together with the results of only h-FTAA in PBS (Figure 2). Comparing the curves with PFFs and only h-FTAA in PBS, an apparent 5-fold increase of fluorescence is obtained at 1000 nM h-FTAA. This shows the usefulness of LCO probes as fluorescent ligands that give a distinct enhanced brightness upon binding to amyloid fibrils, concomitant with the elongated lifetime [29]. By simulating the binding curve, further details on the binding sites of a fluorescent ligand can be assessed, for instance, the binding dissociation constant (K_d_). Heptameric h-FTAA has been reported to have a dissociation constant of 4.8 nM to recombinant prion protein fibrils in a PBS buffer at pH 7.4 [30]. The pentameric LCO p-FTAA has a dissociation constant (K_d_) of 16 nM for insulin fibrils at pH 7.4 [31].

Assuming a one- or a two-site model in the simulation gives similar appearances (Figure 2, dashed red and green curves), so it is hard to deduce the detailed number of binding sites per αsyn moiety in the fibril. However, the simulations set the limit for the effective binding at lower h-FTAA concentrations with a K_d_ < 100 nM and with a quantum efficiency of h-FTAA in the primary binding site being approximately 30–40%, i.e., similar to the case of h-FTAA in ethanol. (More details on the simulations are presented in Figure S4 along with population analysis of free ligands and ligands bound to fibrils). From the binding curve analysis, it can also be concluded that, at concentrations above approximately 1500 nM (Figure S4), the binding sites are filled, and the emission of free ligand starts to dominate the spectral features (see also Figure 1A at 4500 nM). At low relative h-FTAA concentrations (<500 nM), essentially all contributed fluorescence comes from PFF-bound ligands, as there is a negligible amount of free ligands, according to the qualitative kinetic analysis (see simulated populations in Figure S4A,B).

**Figure 3:**
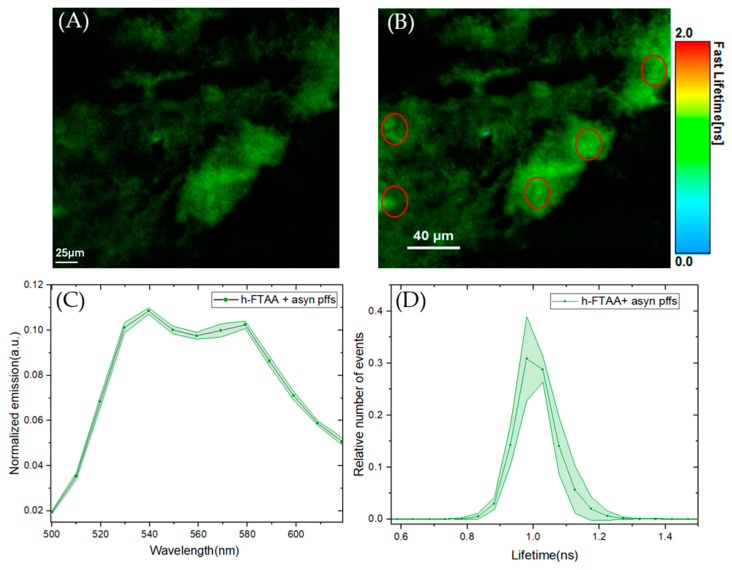
Hyperspectral imaging and fluorescence lifetime measurements of PFFs stained with 500 nM h-FTAA. (**A**) Representative fluorescence image and (**B**) False-color coded FLIM image of PFFs stained with h-FTAA. The sample was excited at 475 nm and the photons were collected in the 500–700 nm range. The color bar to the right represents the lifetime ranging from 0 ns to 2 ns. (**C**) Spectral analysis of h-FTAA when it is bound to PFFs, showing emission maxima at approximately 540 nm and 580 nm. The five ROIs (red) used to record the emission spectra are shown in (**B**). (**D**) Fluorescence decay time distribution recorded from the FLIM image using the same ROIs (red) that were selected for the spectral analysis in (**C**). The shaded regions in the plots represent the standard deviation.

To gain more insight into the fibrillar structure and to optimize the microscope’s spectral settings, 1 μM of the PFFs were stained with 500 nM h-FTAA and then analyzed using hyperspectral microscopy, including fluorescence lifetime imaging microscopy (FLIM). The confocal microscopy image in Figure 3A shows the fibrillar structure of the h-FTAA-stained PFFs, with some mesh-like/cloudy appearance. Upon exciting the sample at 475 nm, the emission spectra were recorded by selecting five different regions of interest (ROIs) within the stained regions. The emission spectrum from each ROI was background corrected and normalized to the total area of each spectrum. This exhibited a homogenous spectral distribution (Figure 3C) with characteristic emission maxima at around 540 and 580 nm, which was also found with the plate reader, shown in Figure 1. The resolved double peak along with red-shifted features are also in agreement with previous data [27] and are more similar to the ethanol case for the solvent examination discussed above. Furthermore, the fluorescence lifetime was recorded for the same images, with the false-color FLIM image depicted in Figure 3B. The fluorescence lifetime distributions corresponding to the same five ROIs, illustrated in Figure 3, are plotted in Figure 3D. The fluorescence decay time distributions from each ROI were normalized to the total of the area of each distribution. The plot in Figure 3D shows a homogeneous distribution which was fitted to a Gaussian function. The parameters associated with this function include the center of the peak, which plausibly represents the lifetime, given as 1.003 ± 0.001 ns, and the full width half maximum (FWHM) of the curve is given as 0.12 ± 0.002 ns. These lifetimes are considerably longer than for h-FTAA, especially in PBS (Table 1), and are also comparable to the literature data [21], indicating a hydrophobic binding site. Taken together, the spectral profiles and FLIM results indicate that the h-FTAA effectively binds to the PFFs.

### 2.3. Characterization of αsyn Protein Expression in HEK293 Cells, Transiently Transfected with Human-A53T or Human WT-αsyn

**Figure 4:**
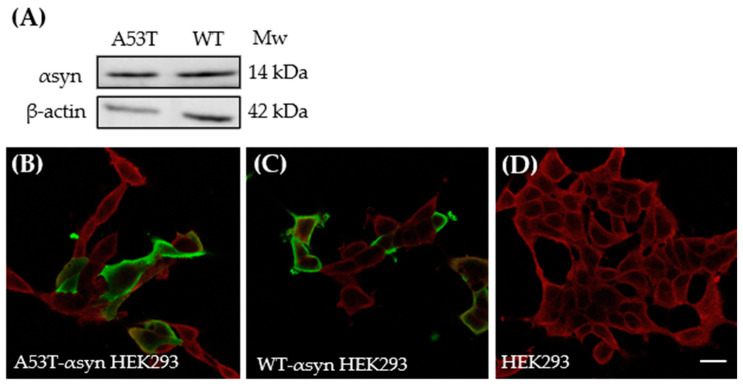
Endogenous αsyn expressed in HEK293 cells after transfection with 4 μg human A53T-αsyn or WT-αsyn. (**A**) Western blot showing αsyn protein bands at approximately 14 kDa in A53T-αsyn and WT-αsyn HEK293 cells which were probed with mouse anti-αsyn antibody Syn211. Representative immunofluorescence images of (**B**) A53T-αsyn, (**C**) WT-αsyn HEK293 cells showing localization of αsyn in cytosol, and (**D**) Untransfected HEK293 cells showing absence of αsyn, when labeled with mouse anti-αsyn antibody Syn211. Scale bar represents 10 µm.

Next, HEK293 cells were transiently transfected with either human-A53T-αsyn (A53T-αsyn-HEK293) or human WT-αsyn (WT-αsyn-HEK293) to induce endogenous expression of αsyn protein. The αsyn protein expression level after transfection was assessed by western blotting using anti-αsyn monoclonal antibody Syn 211. The analysis showed that human A53T-αsyn or human WT-αsyn protein was expressed in the HEK293 cells. Notably, monomeric αsyn protein bands could be detected at approximately 14 kDa in HEK293 cells transfected with A53T-αsyn and WT-αsyn (Figure 4A). Furthermore, the transfected cells were stained for αsyn using the Syn 211 antibody. In addition, a plasma membrane marker, CellMask Deep Red, was added as a co-stain dye to ascertain the localization of αsyn within the cells and subsequently, the cells were imaged using confocal laser scanning microscope. As shown in Figure 4B,C, αsyn native protein (green) in the transfected cells was primarily localized in the cytosol. As expected, Figure 4D shows no expression of αsyn in the control HEK293 cells.

Furthermore, to assess the efficiency of transfection, the transfected cells were stained for αsyn using Syn 211 and a secondary antibody conjugated to Alexa Fluor 647. The fluorescence emission from Alexa Fluor 647 was used for selecting transfected cells using fluorescence-activated cell sorting (FACS). Untransfected HEK293 cells were used for gating A53T-αsyn-HEK293 and WT-αsyn-HEK293 cells (Figure S5). The transfection efficiency for cells transfected with A53T-αsyn was found to be around 21% while for those transfected with WT-αsyn it was about 25%. Conclusively, these results demonstrate that the HEK293 cells transfected with either human A53T-αsyn or human WT-αsyn expressed αsyn protein.

### 2.4. Hyperspectral Imaging and FLIM of αsyn Aggregates in HEK293 Cells

**Figure 5:**
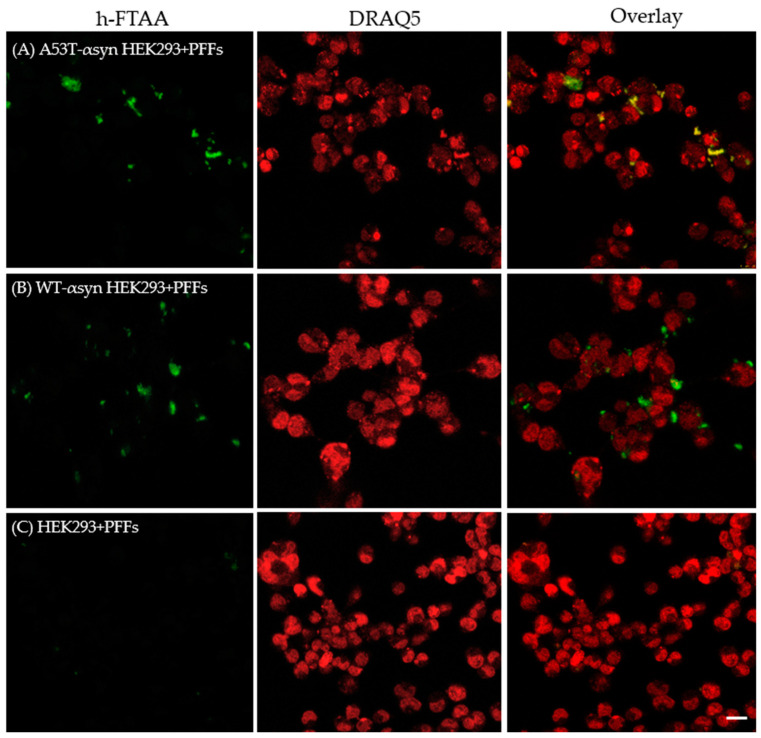
Representative fluorescence images of HEK293 cells expressing (**A**) A53T-αsyn or (**B**) WT-αsyn, seeded with 500 nM human-αsyn PFFs and stained with 1 μM h-FTAA (green) and 5 µM DRAQ5 (red). (**C**) Untransfected HEK293 cells were also exposed to PFFs, showing minimal fluorescence from h-FTAA. Scale bar represents 10 μm.

Furthermore, to understand more about the spectral and lifetime distributions of h-FTAA binding to αsyn aggregates in cells, HEK293 cells expressing A53T or WT-αsyn were exposed to 500 nM of αsyn PFFs in a lipofectamine-mediated transfection. Subsequently, the amyloid aggregates within these cells were visualized by staining them with 1 µM of h-FTAA after three days of initial fibril exposure. Eventually, the aggregates formed within the cells were assessed using hyperspectral imaging and fluorescence lifetime microscopy. Representative fluorescence images of h-FTAA-stained αsyn amyloid aggregates (green) in cells expressing A53T or WT-αsyn, are shown in Figure 5A,B. Notably, h-FTAA exhibited enhanced fluorescence when bound to the aggregates in HEK293 cells expressing A53T or WT, with minimal background interference. In contrast, in the control experiment, the untransfected HEK293 cells showed minimal fluorescence from h-FTAA when seeded with PFFs (Figure 5C). Given that the endogenous expression levels of WT-αsyn and A53T-αsyn in HEK293 cells were comparable (Figure S5), visual inspection of the h-FTAA-stained regions showed that A53T-αsyn-HEK293 cells exhibited a higher degree of aggregation compared to WT-αsyn-HEK293 cells. Since the A53T mutated form of αsyn is prone to show enhanced aggregation [33] when compared to other αsyn variants, the enhanced aggregation observed here lies in line with the literature reports.

**Figure 6:**
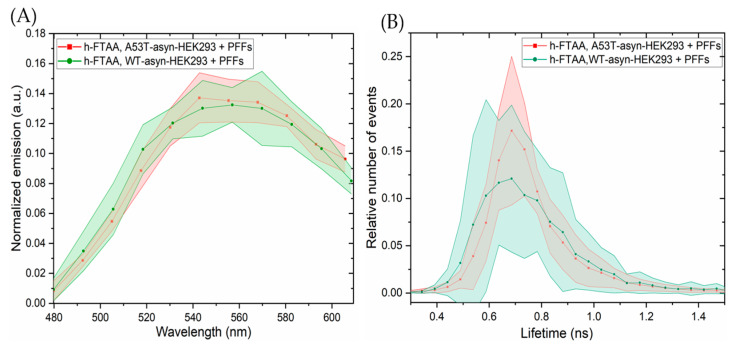
Representative spectral analysis and lifetime distributions of h-FTAA binding to aggregates in A53T-αsyn-HEK293 and WT-αsyn-HEK293 cells. The samples were excited at 475 nm. (**A**) Emission spectra and (**B**) lifetime distributions of h-FTAA binding to aggregates in A53T-αsyn (red) and WT-αsyn-HEK293 (green) cells. Shaded areas correspond to the standard deviation of 5 ROIs.

Next, the fluorescence emissions and lifetime distributions from the induced h-FTAA-stained αsyn aggregates were assessed by exciting h-FTAA using a white light laser tuned at 475 nm. The emission spectra were constructed by selecting different ROIs within the stained regions in the cells and subtracting the emission spectra from the background. In order to compare the emission scans from different experimental setups, the emission spectrum acquired from different ROIs was normalized to the total area of each spectrum. The experiments were run in duplicates as four independent experiments and five ROIs were drawn from each experiment for analysis. When looking at the spectral analysis of the h-FTAA-stained aggregates in Figure 6A, the emission maxima of h-FTAA were observed to be approximately at 540 and 580 nm, showing similar correlations to earlier spectral observations from Section 2.1 and Section 2.2. The emission profiles of h-FTAA (Figure 6A), when bound to the aggregates in A53T-αsyn (red) or WT-αsyn HEK293 cells (green), showed a similar trend. Furthermore, the FLIM images were recorded (Figure S6) for the same set of experiments and the lifetime distributions analyzed from the same five ROIs that were used for the spectral analysis are presented in Figure 6B. The fluorescence decay time distributions of h-FTAA bound to aggregates in A53T-αsyn or WT-αsyn HEK293 cells were fitted to a Gaussian function, yielding the same peak values, which represent lifetime, but with varying peak widths for both cell types. The fitted parameter for the lifetime was 0.71 ± 0.005 ns. The widths, likely representing the standard deviation, are 0.205 ± 0.01 ns for A53T-αsyn-HEK293 cells and 0.3 ± 0.011 ns for WT-αsyn-HEK293 cells. These results verified a considerably longer lifetime than observed for only h-FTAA in the solvents, but not to the extent of the lifetime observed in pristine PFFs (Figure 3). The broader lifetime distribution may indicate the occurrence of more complex interactions between h-FTAA bound to αsyn fibrils in the cell model in terms of interactions of the αsyn fibrils with various cellular components and exposure of the ligands to different solvent surroundings.

**Figure 7:**
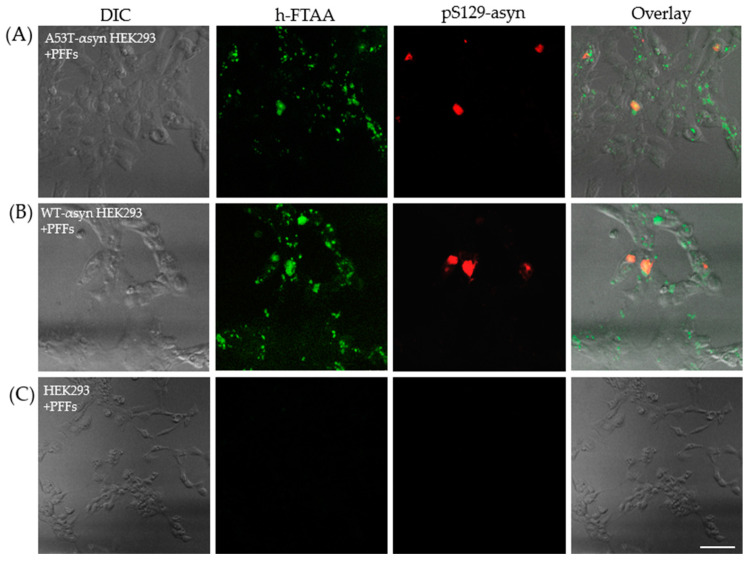
Representative differential interference contrast (DIC) and confocal microscopy images showing h-FTAA-stained (green), pS129-probed αsyn aggregates (red) in (**A**) A53T-αsyn-HEK293 cells and (**B**) WT-αsyn-HEK293 cells. The samples were excited at 475 nm and 650 nm, respectively. (**C**) Untransfected HEK293 cells, also seeded with PFFs, show no fluorescence from h-FTAA or the anti-αsyn pS129 antibody, indicating absence of pS129-positive αsyn aggregates. Scale bar represents 25 µm.

One of the major pathological molecular markers of αsyn aggregation in synucleinopathies is its post-translational modification wherein it majorly undergoes phosphorylation at serine-129 (pS129) [34]. In normal physiology, the expression of αsyn in the brain involves minimal post-translational modification. This distinction helps in identifying αsyn aggregates in pathological conditions compared to the native protein [35]. There are many antibodies available for detecting pS129-αsyn in brain extracts and in cellular models of PD and other related synucleinopathies [36,37]. In this regard, the A53T-αsyn-HEK293 and WT-αsyn-HEK293 cells exposed to PFFs were probed with an anti-αsyn pS129 antibody to identify pS129-positive aggregates. This served as one of the control experiments, substantiating the presence of induced aggregates which are phosphorylated as opposed to the initial αsyn seeds (which are not phosphorylated) in the seeding experiments. The cells were probed with anti-αsyn pS129 antibody followed by staining with a secondary antibody, conjugated to Alexa Fluor 647. Subsequently, the cells were stained with 500 nM of h-FTAA, and the hyperspectral imaging, including FLIM, was recorded for the h-FTAA-stained, pS129-probed aggregates to check whether there were any changes in the spectral or lifetime distributions for the same. Representative confocal images illustrating pS129-positive aggregates (red) with an overlap of h-FTAA staining (green) are presented in Figure 7A,B. Notably, the untransfected HEK293 cells in Figure 7C, which were also exposed to PFFs, did not exhibit a fluorescence signal from h-FTAA or the pS129 antibody labeling, confirming the absence of phosphorylated aggregates. It is also evident that h-FTAA staining always occurs concomitantly with the pS129 antibody, but not vice versa. This might indicate that not all of the fibrils formed from seeding are phosphorylated, and/or that the h-FTAA ligand is more promiscuous to binding αsyn aggregates at different stages of the fibrillation process. The latter will be further elaborated on in Section 3.

**Figure 8:**
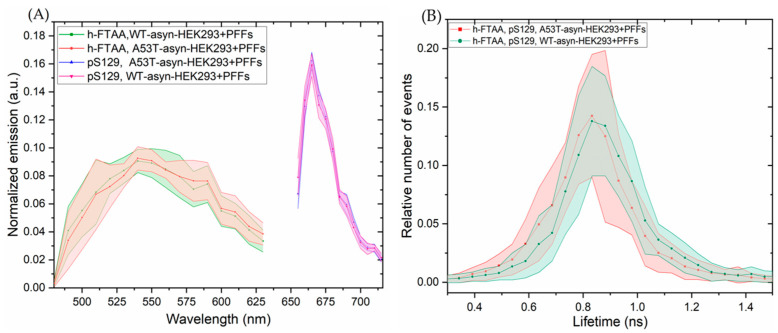
Representative spectral analysis and lifetime distributions of h-FTAA-stained, pS129-labeled aggregates in A53T-αsyn-HEK293 and WT-αsyn-HEK293 cells. The samples were excited at 475 nm and 650 nm for h-FTAA and Alexa Fluor 647, respectively. (**A**) Emission spectra and (**B**) life-time distributions for h-FTAA-stained, pS129-labeled aggregates in A53T-αsyn-HEK293 and WT-αsyn-HEK293 cells. Shaded areas correspond to the standard deviation of 5 ROIs.

The emission spectra were recorded for h-FTAA, when binding to pS129-positive αsyn aggregates, by exciting the sample at 475 nm. The ensuing spectral profiles for h-FTAA in A53T-αsyn-HEK293 (red shaded region) or WT-αsyn-HEK293 (green shaded region) are presented in Figure 8A. The shaded regions in the respective emission spectrum represent the standard deviation from five ROIs, based on three independent experiments. The emission maxima for h-FTAA from pS129-labeled aggregates are approximately at 540 and 570 nm, which is similar to the previous observation (Figure 6A). The emission spectrum for the αsyn-pS129 antibody, labeled with the Alexa Fluor 647 secondary antibody in A53T-αsyn-HEK293 and WT-αsyn-HEK293, was recorded by exciting the sample at 647 nm. The spectral analysis of the pS129-positive αsyn aggregates in Figure 8A shows a homogeneous distribution, with an emission maximum for Alexa Fluor 647 at approximately 665 nm. Moreover, h-FTAA and Alexa Fluor 647 are spectrally well separated due to their individual staining of the aggregates, but they still overlap when visually inspected (Figure 7A,B).

Notably, when the fluorescence lifetime distributions for the same h-FTAA-stained, pS129-positive aggregates were fitted to a Gaussian function (Figure 8B and Figure S7), the resulting parameters indicated possible lifetime values of 0.83 ± 0.0031 ns for A53T-αsyn-HEK293 and 0.86 ± 0.0028 ns for WT-αsyn-HEK293. Both had similar peak widths of ~0.26 ± 0.0006 ns. These lifetime values, observed for A53T-αsyn and WT-αsyn-HEK293 cells, are slightly longer than those reported in previous observations (see Figure 6B), however, with a considerable overlap of the statistical lifetime distributions.

## 3. Discussion

h-FTAA displays a conformational-dependent spectral shift when bound to various disease-associated protein deposits, including αsyn deposits [21,27], whereas other oligothiophene ligands appear to be more selective towards non-αsyn protein pathologies. Hence, the h-FTAA ligand may be particularly valuable for in vitro characterization of αsyn PFFs and induced αsyn aggregates. To our knowledge, in vitro spectral characterization of αsyn–h-FTAA binding has not been carried out before. Therefore, it was essential to first assess the photophysical properties of h-FTAA alone in common solvents, as well as to validate the binding efficiency of h-FTAA to αsyn PFFs.

When assessing the photophysical properties of h-FTAA in solvents with different polarities, the excitation spectra for h-FTAA did not show any variation; however, the emission spectrum in ethanol was slightly red-shifted, suggesting that the excited state of h-FTAA was likely of higher polarity or more stabilized in ethanol, compared to other polar solvents. Moreover, the QY for h-FTAA in ethanol was also observed to be around 40% indicating that the ligand increased the QY in a more nonpolar environment. Strikingly, a lower QY was calculated in PBS compared to other polar solvents. This finding aligns with the minimal background interference that was noted when untransfected HEK293 cells seeded with PFFs were stained with h-FTAA. The ligand binding to fibrillar aggregates is also more complex for the longer oligothiophenes since the π-conjugated chain can be stabilized in a more or less planar configuration. The extended planar conformation of oligothiophenes usually manifests itself in a red-shift as the conjugated framework then has a lower optical bandgap [29,38]. Hence, conformational changes can also counteract the well-known polarity effects for shorter fluorescent ligands that usually yields blue-shifted spectra in the hydrophobic fibril binding sites. The combination of spectral and time-resolved fluorescence together thus allows for a more accurate interpretation of the binding, as shown here.

In the next step, the photophysical properties of h-FTAA binding to PFFs were examined. The hyperspectral imaging gave h-FTAA emission spectrum in a range similar to that observed in polar solvents, particularly ethanol. Furthermore, the decay time for h-FTAA when it was bound to PFFs was observed to be around 1.003 ± 0.001 ns, which was relatively longer than that detected in ethanol and methanol. The difference in lifetime profile observed for h-FTAA, when bound to PFFs compared to its profile in solvents, reflects the changing chemical environment possibly around the binding pocket of h-FTAA, which results in a characteristic lifetime value around 1 ns. The lifetime observed here corroborates to the similar lifetime values observed for in vitro αsyn fibrils formed in different buffer conditions [39].

After validation of h-FTAA’s binding efficacy to αsyn PFFs, cell experiments were performed. In order to understand the potential cellular impact on aggregate morphology, both A53T-αsyn and WT-αsyn HEK293 cells were seeded with PFFs and stained with h-FTAA. The emission spectra of h-FTAA binding to the induced aggregates displayed maxima with broad peaks around 540 and 580 nm, which was similar to the observations of h-FTAA binding to PFFs and in polar solvents. Drawing comparisons with the existing literature, h-FTAA exhibits double peaks at approximately 545 nm and 590 nm, showing a red-shifted spectral shift when it binds to prion deposits in mice brain tissue sections infected with two distinct prion strains [21]. In another study, h-FTAA staining could detect and spectrally differentiate distinct αsyn assemblies in transgenic mouse models of MSA, giving similar emission profiles, as observed in the cells in [16]. These in vivo studies, taken together with our in vitro investigation of h-FTAA binding to αsyn aggregates, leads us to speculate that it could be likely that the binding pocket of h-FTAA might be very well exposed to the hydrophobic environment of the aggregates in such a way that probably stabilizes a more extended excited state, hence yielding a characteristic red-shifted shoulder of the emission spectrum [29,38].

Furthermore, FLIM was also assessed for the h-FTAA-stained aggregates in WT-αsyn and A53T-αsyn HEK293 cells. The fluorescence decay time of a ligand is highly sensitive to its extrinsic environment, and this in turn reflects on the conformation of the molecule to which it binds. h-FTAA is shown to exhibit different fluorescence decay times when it binds to αsyn inclusions in brain tissue sections of PD and MSA patients [17]. Moreover, the fluorescence decays were found to be distinct for morphotypes of prion inclusions in the brain tissue of mice infected with distinct prion strains [21]. In this study, the fluorescence decay times of h-FTAA, when bound to the induced intracellular aggregates in WT-αsyn and A53T-αsyn-HEK293, was found to be to be shorter than the decay time of h-FTAA when bound to PFFs. This difference in lifetime parameters possibly reflects a change in the solvent or chemical environment around h-FTAA, which can lead to complex interactions between the ligand and the aggregates, thereby causing a conformational-specific shift of lifetime. The results thereby indicate a conformational difference between PFFs and the induced αsyn aggregates in the HEK293 cells. The fluorescence lifetime values examined for h-FTAA binding to the aggregates in the cells seem to align with lifetime values observed for h-FTAA in ethanol, suggesting a more hydrophobic milieu around h-FTAA in this case.

For validation purposes, the WT-αsyn and A53T-αsyn-HEK293 cells exposed to PFFs were stained with h-FTAA and double labeled with an antibody specific for pS129-αsyn. The result showed some overlap of h-FTAA-stained regions with the antibody staining of pS129-αsyn. However, h-FTAA did not show complete co-localization with the pS129 antibody, but the opposite was always observed as h-FTAA emissions overlapped onto the pS129 antibody. It is probable that the h-FTAA ligand is more sensitive to binding to a wide range of αsyn morphotypes, including premature or intermediate αsyn conformers like oligomers and protofibrils. This has been observed in the case of h-FTAA binding to early oligomeric aggregates of Abeta [15,27]. This can be a limiting factor when staining with conventional antibodies that detect only mature aggregates after extensive phosphorylation. An increased sensitivity to a multitude of morphotypes for the h-FTAA ligand anticipates, on the other hand, more complex spectral and temporal profiles. Although the fluorescence lifetime values of h-FTAA-stained, pS129-positive aggregates in A53T-αsyn and WT-αsyn-HEK293 cells appeared to be slightly shifted towards a longer lifetime, we consider the measured differences to be too small to draw decisive conclusions. Further examination of the detailed fibrillation process for various αsyn strains seems necessary to resolve more details of this and related issues.

## 4. Materials and Methods

### 4.1. Materials

Recombinant human αsyn PFFs was obtained from DANDRITE, the Danish Research Institute of Translational Neuroscience & Department of Biomedicine, Aarhus University, Aarhus, Denmark. pcDNA 3.1 plasmids encoding human A53T-αsyn or WT-αsyn were a kind gift from Dr. Michel Goedert at MRC Laboratory of Molecular Biology, Cambridge, UK. h-FTAA was acquired from the Department of Physics, Chemistry and Biology, Linkoping University, Linkoping, Sweden. DRAQ5, Cell Mask Deep Red, anti-αsyn monoclonal antibody (Syn 211) were purchased from Thermo Fisher Scientific (Oslo, Norway). Anti-αsyn pS129 antibody (EP1536Y) was purchased from Abcam (Cambridge, UK).

### 4.2. Photophysical Measurements of h-FTAA in Solvents

Steady state fluorescence measurements were carried out using a PTI Quantamaster 8075-22 system (Horiba Jobin Yvon, France) equipped with Double Mono 300 spectrometer chambers for both excitation and emission. A Hamamatsu R928 PMT was used for detection in the range of 185–950 nm. A OB-75X (75W Xenon arc lamp) was used as light source. Data acquisition and basic data-handling were carried out with the Felix Data Analysis software (v. 4.2.2) and further processed and presented using Origin Pro 2020 (v. 9.7.0.188). Steady state absorption spectra were recorded using a Shimadzu UV-1601PC spectrophotometer (Shimadzu Co., Kyoto, Japan). Time-resolved fluorescence decays were recorded using an IBH time-correlated single photon counting (TCSPC) 5000 M spectrometer system (HORIBA Jobin Yvon IBH Ltd., Glaskow, UK) with a 1 nm resolved emission monochromator. The system was equipped with a IBH TBX-04D picosecond photon detection module and the sample was excited using an IBH LED operating at 455 nm. The measured decay-trace was analyzed using deconvolution fitting with the IBH Data Station v. 2.1 and IBH DAS 6 (v. 6.1) softwares and presented using Origin Pro 2020 software (v. 9.7.0.188).

All measurements were performed with 10 mm quartz cuvettes (Hellma Precision). Spectroscopic grade methanol (Sigma-Aldrich, Merck Life-Sciences, Oslo, Norway) and ethanol (VWR Chemicals, Rosny-sous-Bois, France) solvents were used.

### 4.3. αsyn Fibril Formation and Transmission Electron Microscopy Analysis

Full-length wildtype human αsyn was expressed in BL21(DE3)-competent cells and purified by ion-exchange and reverse phase chromatography, as previously described [40,41]. To generate pre-formed fibrils (PFFs), 4 mg/mL of soluble monomeric αsyn in PBS (pH 7.4, Gibco, Thermo Fisher Scientific, Roskilde, Denmark) was incubated at 37 °C for 72 h with continuous shaking (1050 rpm, Eppendorf Thermotop, Sigma-Aldrich, Søborg, Denmark). Fractions were tested for ThT fluorometry and sedimentation analysis to validate amyloid structure and insolubility, as previously described [41]. The validated PFFs were harvested by centrifugation at 15,600× *g* for 30 min into pellet insoluble fibrils, then resuspended in PBS to a concentration of 1 mg/mL, determined using a Pierce BCA protein assay (Thermo Fisher Scientific). The PFFs were then sonicated for 20 min with 30 ms pulses, followed by 70 ms breaks at 30% power using a Branson (Merck Life Science, Søborg, Denmark) SFX250 Sonifier equipped with a 1” cup horn (Branson; 101-147-046) and stored at −80 °C.

PFFs were thawed and imaged using TEM. Here, the PFF stock solution (70 μM on a monomer basis) sample was applied to the TEM grid to adsorb for 2 min (400 mesh copper grids CARBON-B, Ted Pella Inc., Redding, CA, USA). The excess sample was botted off with filter paper, the grid was washed with milli-Q water, and was negative stained with 2% uranyl acetate for 30 s and grids were blotted dry and dried in air. Imaging was performed using a Jeol JEM1400 Flash TEM instrument (JEOL Nordic AB, Hammerbacken, Sweden) with images taken at 10,000× magnification.

### 4.4. Emission of αsyn PFFs Together with h-FTAA and Simulation of Binding Curves

For spectral assessment and binding curves, the αsyn PFFs were diluted to 1 µM (on a monomer basis) in PBS and mixed with varying concentrations of h-FTAA ranging between 0 and 4500 nM. Following this, they were incubated overnight at room temperature in a black 96-well plate with a clear bottom (Corning 8085, Fisher Scientific). The excitation and fluorescence spectra were measured using a Tecan Saphire 2 plate reader using excitation spectra of 400–520 nm (emission 550 nm) and emission spectra of 500–700 nm (450 nm excitation) with 5 nm steps and slits. The one and two site-binding models, used to simulate binding kinetics, were developed to calculate the relative abundance of free ligands, empty protein sites, as well as the protein filled in the one- and two-sites (see [32] for further details).

### 4.5. In Vitro PFFs-h-FTAA Characterization Using Hyperspectral Imaging and FLIM

PFFs at 1 µM were mixed with h-FTAA at 500 nM. Microscopy slides were prepared by adding 2 µL of the stained PFFs. The samples were dried and then covered with a coverslip. The pre-stained PFFs were assessed for spectral features and FLIM using a Leica SP8 (Ortomedic, Leica Microsystems, Oslo, Norway) with single molecule detection and multiphoton laser confocal microscope. PicoQuant’s SymphoTime 64-bit version was used for FLIM measurement and data analysis.

### 4.6. Cell Cultivation

The HEK293 cells originate from fetal kidney’s epithelial cells that are immortalized by transforming human kidney cells with adenovirus type 5 DNA [42]. These were grown in Eagle’s Minimum Essential Medium (EMEM) (ATCC^®^ 30-2003, Cambridge, UK) and supplemented with 10% Fetal Bovine Serum (FBS) (Sigma Aldrich, F-7524, Merck Life Sciences, Norway). The HEK293 cells were incubated at 37 °C in a humidified chamber with 5% CO_2_. Upon reaching 80–90% confluency, they were regularly sub-cultivated. The cells were prepared for sub-cultivation by removing the growth medium and washing them with PBS (Sigma Aldrich) to get rid of traces of the old medium. After detaching the cells from the culture surface using a 0.25% Trypsin-EDTA solution (Sigma Aldrich), they were incubated at 37 °C for 3–5 min in a humidified chamber with 5% CO_2_. The trypsinized cells were then added to the EMEM growth medium, supplemented with 10% FBS and mixed thoroughly to prevent cell aggregation. The cell suspension was then transferred to a test tube and centrifuged (1500 rpm, 5 min). A fraction of the cell suspension of known concentration was prepared and used to seed two new flasks (2 × 10^6^ cells in a T-75 or 0.5 × 10^6^ cells in a T-25 flask). Appropriate volume of fresh EMEM growth medium, supplemented with 10% FBS, was added to the new flasks followed by incubating the sub-cultivated cells (37 °C, 5% CO_2_). Apart from sub-culturing, the cells were washed (PBS) and replenished with fresh growth medium, supplemented with 10% FBS, once a week.

### 4.7. Transfection with A53T or WT-αsyn

HEK293 cells were plated at a density of 300,000 cells in 6-well plates, and transfected with 4 µg of pcDNA 3.1-plasmid containing A53T-αsyn or WT-αsyn, using Lipofectamine 2000 (Invitrogen, Oslo, Norway). The cells were harvested after 24 h of transfection.

### 4.8. Seeding with αsyn PFFs

Transfected cells were plated in 8-well plates (µ-slide ibidi) at a plating density of 5 × 10^5^ cells/mL and incubated overnight (37 °C, 5% CO_2_). To expose the cells to PFFs, the fibrils were sonicated for 5 min using Bandelin Sonorex at 35 kHz. The PFFs were mixed with Opti-MEM (Gibco) and incubated for 5 min at room temperature. Simultaneously, the lipofectamine 2000 (Invitrogen) was mixed with Opti-MEM at 1:10 dilution and was incubated for 5 min at room temperature. The lipofectamine–Opti-MEM mixture was added to the PFFs–OptiMEM mixture at a 1:1 ratio, incubated for 10 min, and then added to the cells at a final concentration of 500 nM. After three hours of incubation, the cells were washed twice with PBS (Gibco) to remove any extracellular fibrils, replenished with fresh medium containing EMEM supplemented with FBS (10%), and were incubated for three days (37 °C, 5% CO_2_).

### 4.9. Staining with h-FTAA, DRAQ5 and Hyperspectral Microscopy

Following three days of incubation, 1 µM of h-FTAA was added to the cells and incubated (1 h) at room temperature. After incubation, the cells were washed with PBS and then stained with DRAQ5 (5 µM). After 10 min of incubation, the cells were rinsed with PBS and imaged using a Leica SP8 with single molecule detection and a multiphoton laser confocal microscope. For measuring and analyzing lifetime distributions, Pico Quant’s SymPhoTime 64-bit version was utilized by exciting the sample at 475 nm for h-FTAA using a pulse laser set at 40 MHz and keeping the laser intensity between 5 and 10%.

### 4.10. FACS for Assessing Transfection Efficiency

Cells were grown in 6-well plates (Corning Costar^®^ Sigma-Aldrich, Merck Life-Sciences, Oslo, Norway) and harvested using a Trypsin-EDTA (0.25%) acidic solution (Sigma-Aldrich, Merck Life-Sciences, Oslo, Norway). Following two washings (PBS), the cells were spun at 1500 rpm and fixed with a Fixation solution (BD Biosciences, Oslo Norway) for 30 min at room temperature. After washing the fixed cells with PermWash (BD Biosciences, Oslo, Norway), they were treated with an anti-αsyn monoclonal antibody (Syn211, Invitrogen), which was diluted 1:2000 in PermWash, and incubated for 2 h. Following one washing (PermWash), the cells were incubated with goat anti-mouse secondary antibody conjugated to Alexa Fluor 647 (Invitrogen), which was diluted 1:500 in PermWash and then incubated for 45 min at room temperature. Following one washing (PermWash), the cells were resuspended in PBS and analyzed using BD FACS Aria II.

### 4.11. Western Blotting

Cells were grown in 35 mm cell-culture dishes (Corning Costar^®^), lysed using a 1× NuPAGE LDS sample buffer, including a sample reducing agent (Invitrogen), on ice with the help of a cell scrapper. The lysates were then transferred to Eppendorf tubes. The protein in the samples was denatured by heating them at 90 °C for 10 min. Samples were further sonicated for 10 min and spun at 1300 rpm for 1 min before loading onto the NuPAGE^TM^ 10% Bis-Tris gel (Invitrogen). A protein molecular weight ladder (Sea blue^TM^ Plus2 Pre-stained Protein Standard, Invitrogen) was added to one of the wells and the samples were run at 200V for 25 min. The protein was separated and then transferred to a nitro-cellulose membrane (Bio-Rad, Oslo, Norway) using the Trans-Blot Turbo transfer system (Bio-Rad). Following protein transfer, the membrane was blocked with 5% non-fat dry skim milk in TBS-Tween and further incubated with an αsyn monoclonal antibody (1:500, Syn 211), diluted in 5% non-fat dry skim milk in TBS-Tween. Following three washes, the blot was incubated with an infrared dye goat anti-mouse secondary antibody (IRDye 680RD, LI-COR Biotechnology, Gmbh, Lincoln, NE, USA) which was diluted 1:10,000 in 1% non-fat dry skim milk in TBS-Tween. After three washes in TBS-Tween, the protein bands were visualized using Chemi Doc Imaging Systems (Bio-Rad).

### 4.12. Immunocytochemistry

Cells were rinsed with PBS and then fixed with paraformaldehyde (4%) for 20 min at room temperature. Non-specific sites were blocked with the blocking buffer (3% BSA in PBS, 0.1% Triton X-100) for 1 h at room temperature. The cells were incubated with primary antibodies overnight (4 °C). Then, they were washed with PBS (3 × 10 min). Subsequently, the cells were treated with a secondary antibody for 1 h at room temperature. Further, the cells were washed with PBS (3 × 10 min). Depending on the experiment, the cells were either incubated with a suitable stain, such as 1× CellMask Deep Red (Invitrogen) for 10 min before imaging, or they were incubated with 1 µM h-FTAA for 1 h at room temperature. After incubation, the cells were washed twice (PBS) and then imaged using a Leica SP8 with single molecule detection and multiphoton laser confocal microscope. The primary antibodies used were anti-αsyn monoclonal antibody (1:500, Syn 211, Invitrogen) and anti-αsyn pS129 antibody (1:2000, EP1536Y Abcam), and the secondary antibodies were goat-anti-mouse-secondary antibody conjugated to Alexa Fluor 488 (1:2000, Invitrogen) and goat-anti-rabbit-secondary antibody conjugated to Alexa Fluor 647 (1:2000, Invitrogen).

## 5. Conclusions

The clinical representation of distinct synucleinopathies is heterogenous and overlapping, especially at the early disease stages. The variable sequence of similar motor and non-motor symptoms across distinct synucleinopathies at the early disease stages has limited diagnosis for more advanced disease stages. Particularly, non-motor symptoms can complicate diagnosis when they dominate the clinical features and precede movement dysfunction. Motor and non-motor symptoms have been associated with the presence of αsyn pathology in the central and peripheral autonomic nervous system, affecting multiple organs. Accumulating evidence indicates an association between different αsyn morphotypes and different subtypes of synucleinopathies. Hence, the assessment of the early subtype-specific diagnosis, which is necessary for reliable prognosis and optimal treatment, was conducted. Here, we utilized an in vitro cell culture model to assess the morphology of αsyn aggregates in a controlled physicochemical environment.

A heptameric LCO, h-FTAA, in combination with spectral imaging, was employed to gauge the chemical environment around the fibrillar aggregates based on the conformation state of the LCO. HEK293 cells, expressing A53T-αsyn and WT-αsyn, were exposed to PFFs and the induced intracellular αsyn aggregates were stained with h-FTAA. The hyperspectral imaging and FLIM analysis of h-FTAA, when binding to the induced intracellular αsyn aggregates, indicated a characteristic, red-shifted spectrum consistent with previous studies. Double-labeling experiments, involving co-staining with a pS129-αsyn specific antibody showed incomplete overlap between pS129-labeled and h-FTAA-stained regions within the cells, probably due to restricted binding of the pS129-αsyn antibody to exclusively mature aggregates, whereas the h-FTAA ligand can also detect other morphologies, as previously observed for amyloid-β aggregates [15,27].

This study demonstrates the use of the h-FTAA ligand to explore different αsyn morphotypes in vitro. This h-FTAA-αsyn aggregate interaction, assessed in a cell culture, could be used as a new model to investigate the conformational variability of αsyn seeds isolated from different tissues from animal models of different synucleinopathies [42,43,44,45]. However, it also suggests that alternative oligothiophene fluorescent ligands are screened for more specific detection of certain αsyn phenotypes, in conjunction with further work on cell models. This approach will be extended to investigate the amyloid seeding capacity onto our cell models by various αsyn strains [46,47], both in vitro and from animal models. The differential diagnosis of distinct synucleinopathies, based on the detection of distinct αsyn morphotypes in gut or skin biopsies or bodily fluids, would be groundbreaking and allow earlier and personalized intervention.

## Figures and Tables

**Table 1:** Photophysical parameters of h-FTAA in various solvents. *τ_i_* denotes lifetime and *B_i_* relative amplitude of two-decay fitting model. For TCSPC ~1.0 μM concentration of h-FTAA was used with λ_ex_ = 455 nm. For emission spectra to determine QY, λ_ex_ = 430 nm.

Solvent	$\tau_{1}(ps)$	$B_{1}(\%)$	$\tau_{2}(ps)$	$B_{2}(\%)$	QY(%)
PBS	332±10	88	788±21	12	6.4±0.45
EtOH	287±20	45	862±9	55	43.3±2.3
MeOH	464±21	71	943±24	29	10.9±0.9

## Data Availability

Data is contained within the article and Supplementary Materials.

## Supplementary Materials

The following supporting information can be downloaded at: https://www.mdpi.com/article/10.3390/ijms252212458/s1.

## References

[1] Peelaerts W., Bousset L., Van der Perren A., Moskalyuk A., Pulizzi R., Giugliano M., Van den Haute C., Melki R., Baekelandt V. (2015). α-Synuclein strains cause distinct synucleinopathies after local and systemic administration. Nature.

[2] Peng C., Gathagan R.J., Covell D.J., Medellin C., Stieber A., Robinson J.L., Zhang B., Pitkin R.M., Olufemi M.F., Luk K.C. (2018). Cellular milieu imparts distinct pathological α-synuclein strains in α-synucleinopathies. Nature.

[3] Strohäker T., Jung B.C., Liou S.-H., Fernandez C.O., Riedel D., Becker S., Halliday G.M., Bennati M., Kim W.S., Lee S.-J. (2019). Structural heterogeneity of α-synuclein fibrils amplified from patient brain extracts. Nat. Commun..

[4] Peelaerts W., Baekelandt V. (2023). ⍺-Synuclein Structural Diversity and the Cellular Environment in ⍺-Synuclein Transmission Models and Humans. Neurotherapeutics.

[5] Holec S.A.M., Woerman A.L. (2021). Evidence of distinct α-synuclein strains underlying disease heterogeneity. Acta Neuropathol..

[6] Li B., Ge P., Murray K.A., Sheth P., Zhang M., Nair G., Sawaya M.R., Shin W.S., Boyer D.R., Ye S. (2018). Cryo-EM of full-length α-synuclein reveals fibril polymorphs with a common structural kernel. Nat. Commun..

[7] Grazia Spillantini M., Anthony Crowther R., Jakes R., Cairns N.J., Lantos P.L., Goedert M. (1998). Filamentous α-synuclein inclusions link multiple system atrophy with Parkinson’s disease and dementia with Lewy bodies. Neurosci. Lett..

[8] Tao Y., Sun Y., Lv S., Xia W., Zhao K., Xu Q., Zhao Q., He L., Le W., Wang Y. (2022). Heparin induces α-synuclein to form new fibril polymorphs with attenuated neuropathology. Nat. Commun..

[9] Hoppe S.O., Uzunoğlu G., Nussbaum-Krammer C. (2021). α-Synuclein Strains: Does Amyloid Conformation Explain the Heterogeneity of Synucleinopathies?. Biomolecules.

[10] Schweighauser M., Shi Y., Tarutani A., Kametani F., Murzin A.G., Ghetti B., Matsubara T., Tomita T., Ando T., Hasegawa K. (2020). Structures of α-synuclein filaments from multiple system atrophy. Nature.

[11] Yang Y., Murzin A.G., Peak-Chew S., Franco C., Garringer H.J., Newell K.L., Ghetti B., Goedert M., Scheres S.H.W. (2023). Cryo-EM structures of Aβ40 filaments from the leptomeninges of individuals with Alzheimer’s disease and cerebral amyloid angiopathy. Acta Neuropathol. Commun..

[12] Outeiro T.F. (2021). Alpha-Synuclein Antibody Characterization: Why Semantics Matters. Mol. Neurobiol..

[13] Kumar S.T., Jagannath S., Francois C., Vanderstichele H., Stoops E., Lashuel H.A. (2020). How specific are the conformation-specific α-synuclein antibodies? Characterization and validation of 16 α-synuclein conformation-specific antibodies using well-characterized preparations of α-synuclein monomers, fibrils and oligomers with distinct structures and morphology. Neurobiol. Dis..

[14] Björk L., Klingstedt T., Nilsson K.P.R. (2023). Thiophene-Based Ligands: Design, Synthesis and Their Utilization for Optical Assignment of Polymorphic-Disease-Associated Protein Aggregates. ChemBioChem.

[15] Nyström S., Psonka-Antonczyk K.M., Ellingsen P.G., Johansson L.B., Reitan N., Handrick S., Prokop S., Heppner F.L., Wegenast-Braun B.M., Jucker M. (2013). Evidence for age-dependent in vivo conformational rearrangement within Aβ amyloid deposits. ACS Chem. Biol..

[16] Torre-Muruzabal T., Van der Perren A., Coens A., Gelders G., Janer A.B., Camacho-Garcia S., Klingstedt T., Nilsson P., Stefanova N., Melki R. (2022). Host oligodendrogliopathy and α-synuclein strains dictate disease severity in multiple system atrophy. Brain.

[17] Klingstedt T., Ghetti B., Holton J.L., Ling H., Nilsson K.P.R., Goedert M. (2019). Luminescent conjugated oligothiophenes distinguish between α-synuclein assemblies of Parkinson’s disease and multiple system atrophy. Acta Neuropathol. Commun..

[18] Becker W. (2012). Fluorescence lifetime imaging—Techniques and applications. J. Microsc..

[19] Esbjörner E.K., Chan F., Rees E., Erdelyi M., Luheshi L.M., Bertoncini C.W., Kaminski C.F., Dobson C.M., Kaminski Schierle G.S. (2014). Direct Observations of Amyloid β Self-Assembly in Live Cells Provide Insights into Differences in the Kinetics of Aβ(1–40) and Aβ(1–42) Aggregation. Cell. Chem. Biol..

[20] Klucken J., Outeiro T.F., Nguyen P., McLean P.J., Hyman B.T. (2006). Detection of novel intracellular O-synuclein oligomeric species by fluorescence lifetime imaging. FASEB J..

[21] Magnusson K., Simon R., Sjölander D., Sigurdson C.J., Hammarström P., Nilsson K.P.R. (2014). Multimodal fluorescence microscopy of prion strain specific PrP deposits stained by thiophene-based amyloid ligands. Prion.

[22] Just M.K., Gram H., Theologidis V., Jensen P.H., Nilsson K.P.R., Lindgren M., Knudsen K., Borghammer P., Van Den Berge N. (2022). Alpha-Synuclein Strain Variability in Body-First and Brain-First Synucleinopathies. Front. Aging Neurosci..

[23] Narhi L., Wood S.J., Steavenson S., Jiang Y., Wu G.M., Anafi D., Kaufman S.A., Martin F., Sitney K., Denis P. (1999). Both Familial Parkinson’s Disease Mutations Accelerate α-Synuclein Aggregation. J. Biol. Chem..

[24] Giasson B.I., Duda J.E., Quinn S.M., Zhang B., Trojanowski J.Q., Lee V.M.-Y. (2002). Neuronal α-synucleinopathy with severe movement disorder in mice expressing A53T human α-synuclein. Neuron.

[25] Sun Y., Hou S., Zhao K., Long H., Liu Z., Gao J., Zhang Y., Su X.-D., Li D., Liu C. (2020). Cryo-EM structure of full-length α-synuclein amyloid fibril with Parkinson’s disease familial A53T mutation. Cell Res..

[26] Vasili E., Dominguez-Meijide A., Flores-León M., Al-Azzani M., Kanellidi A., Melki R., Stefanis L., Outeiro T.F. (2022). Endogenous Levels of Alpha-Synuclein Modulate Seeding and Aggregation in Cultured Cells. Mol. Neurobiol..

[27] Klingstedt T., Aslund A., Simon R.A., Johansson L.B., Mason J.J., Nyström S., Hammarström P., Nilsson K.P. (2011). Synthesis of a library of oligothiophenes and their utilization as fluorescent ligands for spectral assignment of protein aggregates. Org. Biomol. Chem..

[28] Rurack K., Spieles M. (2011). Fluorescence Quantum Yields of a Series of Red and Near-Infrared Dyes Emitting at 600–1000 nm. Anal. Chem..

[29] Gustafsson C., Shirani H., Leira P., Rehn D.R., Linares M., Nilsson K.P.R., Norman P., Lindgren M. (2021). Deciphering the Electronic Transitions of Thiophene-Based Donor-Acceptor-Donor Pentameric Ligands Utilized for Multimodal Fluorescence Microscopy of Protein Aggregates. ChemPhysChem.

[30] Herrmann U.S., Schütz A.K., Shirani H., Huang D., Saban D., Nuvolone M., Li B., Ballmer B., Åslund A.K.O., Mason J.J. (2015). Structure-based drug design identifies polythiophenes as antiprion compounds. Sci. Transl. Med..

[31] Taylor C.G., Meisl G., Horrocks M.H., Zetterberg H., Knowles T.P.J., Klenerman D. (2018). Extrinsic Amyloid-Binding Dyes for Detection of Individual Protein Aggregates in Solution. Anal. Chem..

[32] Sundnes M.S.P., Lindgren M., Mohite G., Hellstrand E., Nyström S., Hammarström P. (2024). The fluorescent amyloid ligand X34 binding to TTR tetramer and TTR fibrils: FRET and binding constants of a sequential two-step process. ChemPhotoChem.

[33] Lee M.K., Stirling W., Xu Y., Xu X., Qui D., Mandir A.S., Dawson T.M., Copeland N.G., Jenkins N.A., Price D.L. (2002). Human α-synuclein-harboring familial Parkinson’s disease-linked Ala-53 → Thr mutation causes neurodegenerative disease with α-synuclein aggregation in transgenic mice. Proc. Natl. Acad. Sci. USA.

[34] Anderson J.P., Walker D.E., Goldstein J.M., de Laat R., Banducci K., Caccavello R.J., Barbour R., Huang J., Kling K., Lee M. (2006). Phosphorylation of Ser-129 Is the Dominant Pathological Modification of α-Synuclein in Familial and Sporadic Lewy Body Disease. J. Biol. Chem..

[35] Fujiwara H., Hasegawa M., Dohmae N., Kawashima A., Masliah E., Goldberg M.S., Shen J., Takio K., Iwatsubo T. (2002). α-Synuclein is phosphorylated in synucleinopathy lesions. Nat. Cell Biol..

[36] Delic V., Chandra S., Abdelmotilib H., Maltbie T., Wang S., Kem D., Scott H.J., Underwood R.N., Liu Z., Volpicelli-Daley L.A. (2018). Sensitivity and specificity of phospho-Ser129 α-synuclein monoclonal antibodies. J. Comp. Neurol..

[37] Lashuel H.A., Mahul-Mellier A.-L., Novello S., Hegde R.N., Jasiqi Y., Altay M.F., Donzelli S., DeGuire S.M., Burai R., Magalhães P. (2022). Revisiting the specificity and ability of phospho-S129 antibodies to capture alpha-synuclein biochemical and pathological diversity. npj Park. Dis..

[38] Sjöqvist J., Linares M., Lindgren M., Norman P. (2011). Molecular dynamics effects on luminescence properties of oligothiophene derivatives: A molecular mechanics–response theory study based on the CHARMM force field and density functional theory. Phys. Chem. Chem. Phys..

[39] Chung C.W., Stephens A.D., Ward E., Feng Y., Davis M.J., Kaminski C.F., Kaminski Schierle G.S. (2022). Label-Free Characterization of Amyloids and Alpha-Synuclein Polymorphs by Exploiting Their Intrinsic Fluorescence Property. Anal. Chem..

[40] Lindersson E., Beedholm R., Højrup P., Moos T., Gai W., Hendil K.B., Jensen P.H. (2004). Proteasomal inhibition by alpha-synuclein filaments and oligomers. J. Biol. Chem..

[41] Ferreira N., Gram H., Sorrentino Z.A., Gregersen E., Schmidt S.I., Reimer L., Betzer C., Perez-Gozalbo C., Beltoja M., Nagaraj M. (2021). Multiple system atrophy-associated oligodendroglial protein p25α stimulates formation of novel α-synuclein strain with enhanced neurodegenerative potential. Acta Neuropathol..

[42] Graham F.L., Smiley J., Russell W.C., Nairn R. (1977). Characteristics of a human cell line transformed by DNA from human adenovirus type 5. J. Gen. Virol..

[43] Holmqvist S., Chutna O., Bousset L., Aldrin-Kirk P., Li W., Björklund T., Wang Z.-Y., Roybon L., Melki R., Li J.-Y. (2014). Direct evidence of Parkinson pathology spread from the gastrointestinal tract to the brain in rats. Acta Neuropathol..

[44] Uemura N., Yagi H., Uemura M.T., Hatanaka Y., Yamakado H., Takahashi R. (2018). Inoculation of α-synuclein preformed fibrils into the mouse gastrointestinal tract induces Lewy body-like aggregates in the brainstem via the vagus nerve. Mol. Neurodegener..

[45] Van Den Berge N., Ferreira N., Gram H., Mikkelsen T.W., Alstrup A.K.O., Casadei N., Tsung-Pin P., Riess O., Nyengaard J.R., Tamgüney G. (2019). Evidence for bidirectional and trans-synaptic parasympathetic and sympathetic propagation of alpha-synuclein in rats. Acta Neuropathol..

[46] Zampar S., Di Gregorio S.E., Grimmer G., Watts J.C., Ingelsson M. (2024). “Prion-like” seeding and propagation of oligomeric protein assemblies in neurodegenerative disorders. Front. Neurosci..

[47] Vaneyck J., Segers-Nolten I., Broersen K., Claessens M.M.A.E. (2021). Cross-seeding of alpha-synuclein aggregation by amyloid fibrils of food proteins. J. Biol. Chem..

