# Supplementary material for: In Vitro Cell Model Investigation of Alpha-Synuclein Aggregate Morphology Using Spectroscopic Imaging
Source: Int J Mol Sci. 2024 Nov 20;25(22):12458. doi: 10.3390/ijms252212458 (PMC11594916; doi:10.3390/ijms252212458)

Supplementary document: Figures S1 – S7. Original Western Blot (last page).

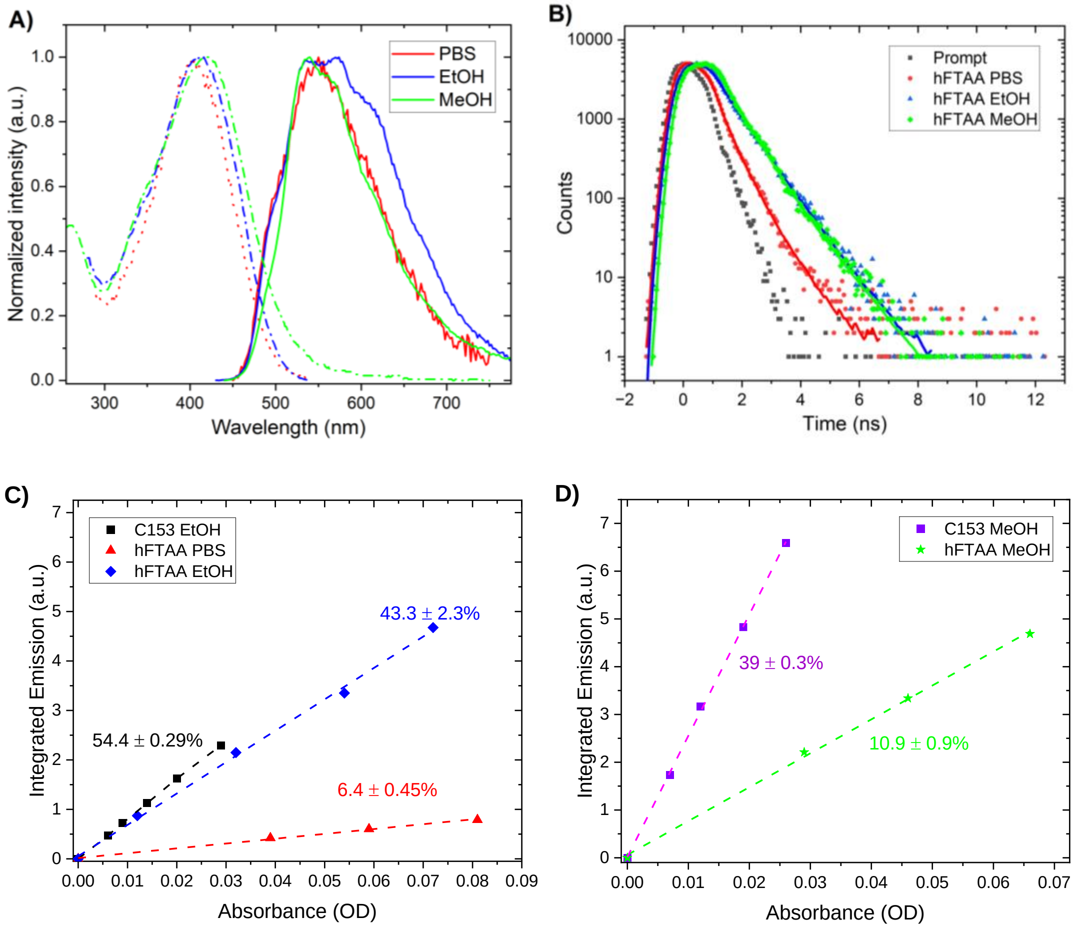

Figure S1. Summary of photophysical characterization of hFTAA in PBS, MeOH and EtOH. Solvent concentrations in the 0.5 – 5 μM range. **A)** Normalized absorption (dashed line) and emission (solid line) spectra (l_{ex} = 430 nm). **B)** TC-SPC decay traces (λ_{ex} = 455 nm). **C)** and **D)** Slope plots for quantum efficiency in various solvents. Coumarin 153 (C153) was used as reference, see [28].

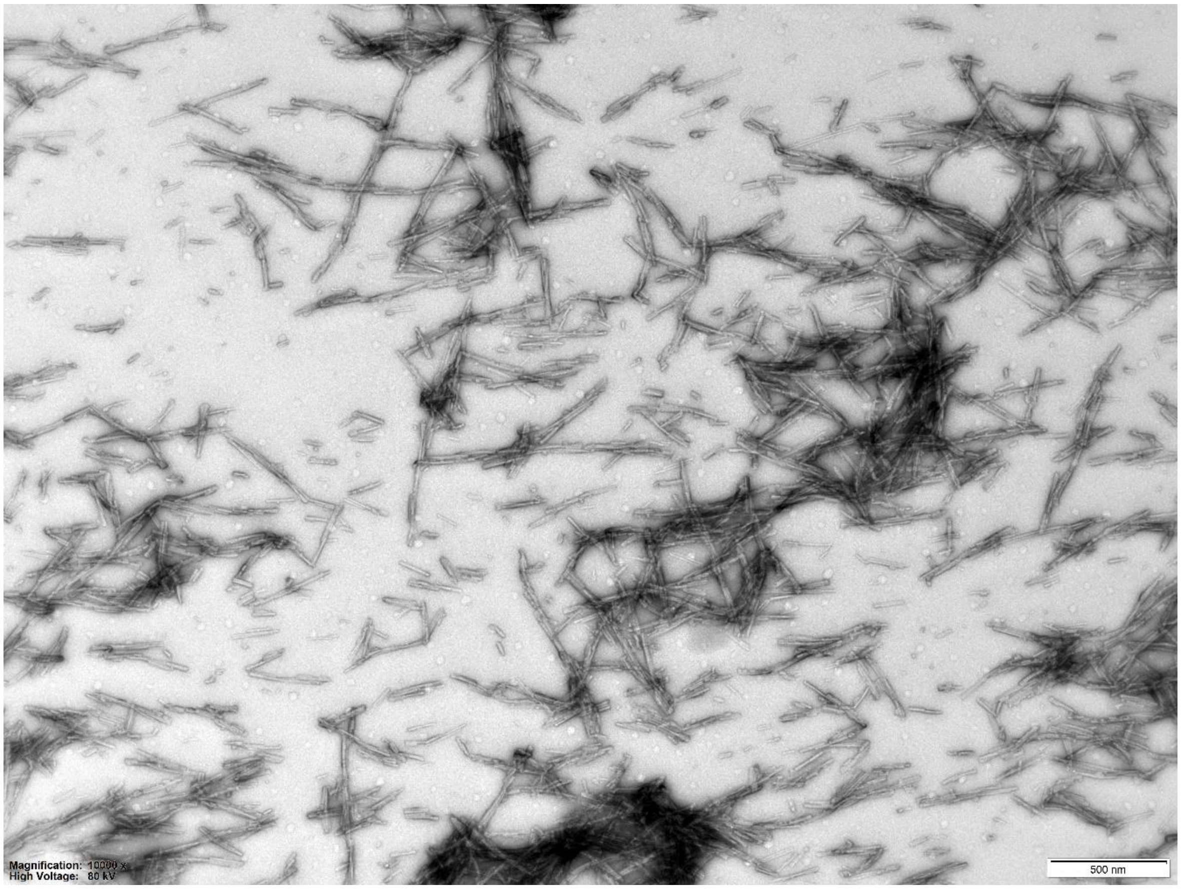

Figure S2. Fibril morphology of human αsyn PFFs by TEM, showing canonical αsyn amyloid fibrils.

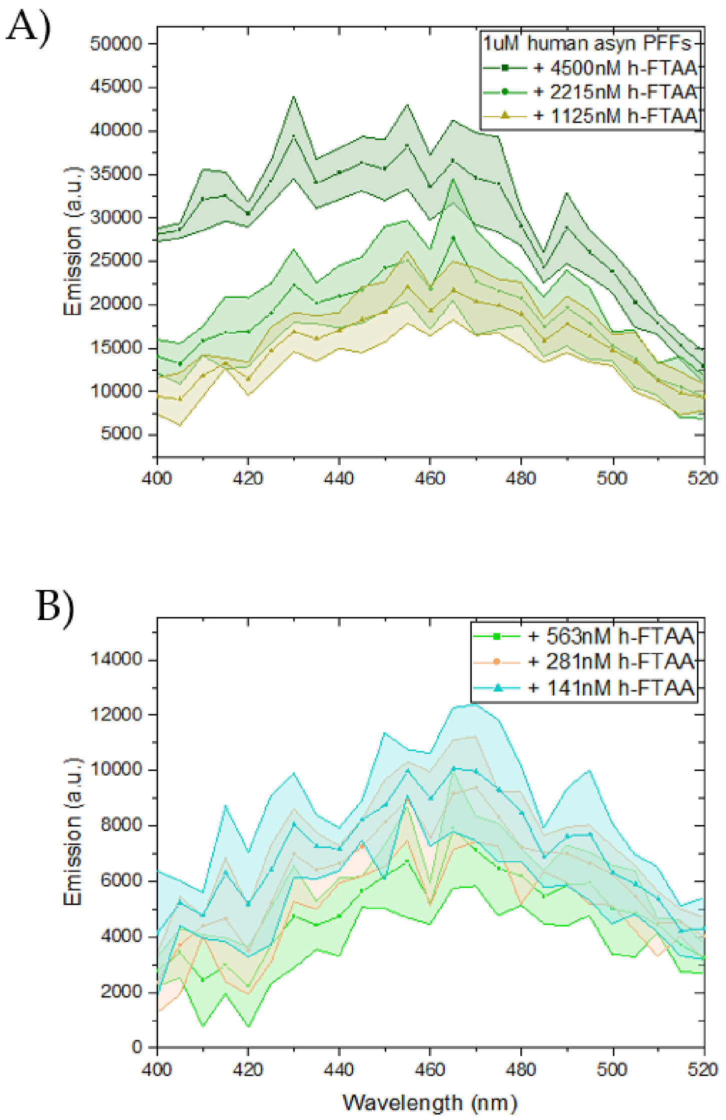

Figure S3: Excitation spectra of h-FTAA together with PFFs by keeping the PFFs fixed at 1uM and varying the concentration of h-FTAA from **A)** 4500nM to 1125nM and **B)** from 563nM to 141 nM. The spectrum at each concentration is background corrected with the excitation spectrum from h-FTAA in PBS. The shaded region of each spectrum represents the triplicates of h-FTAA loading at each concentration.

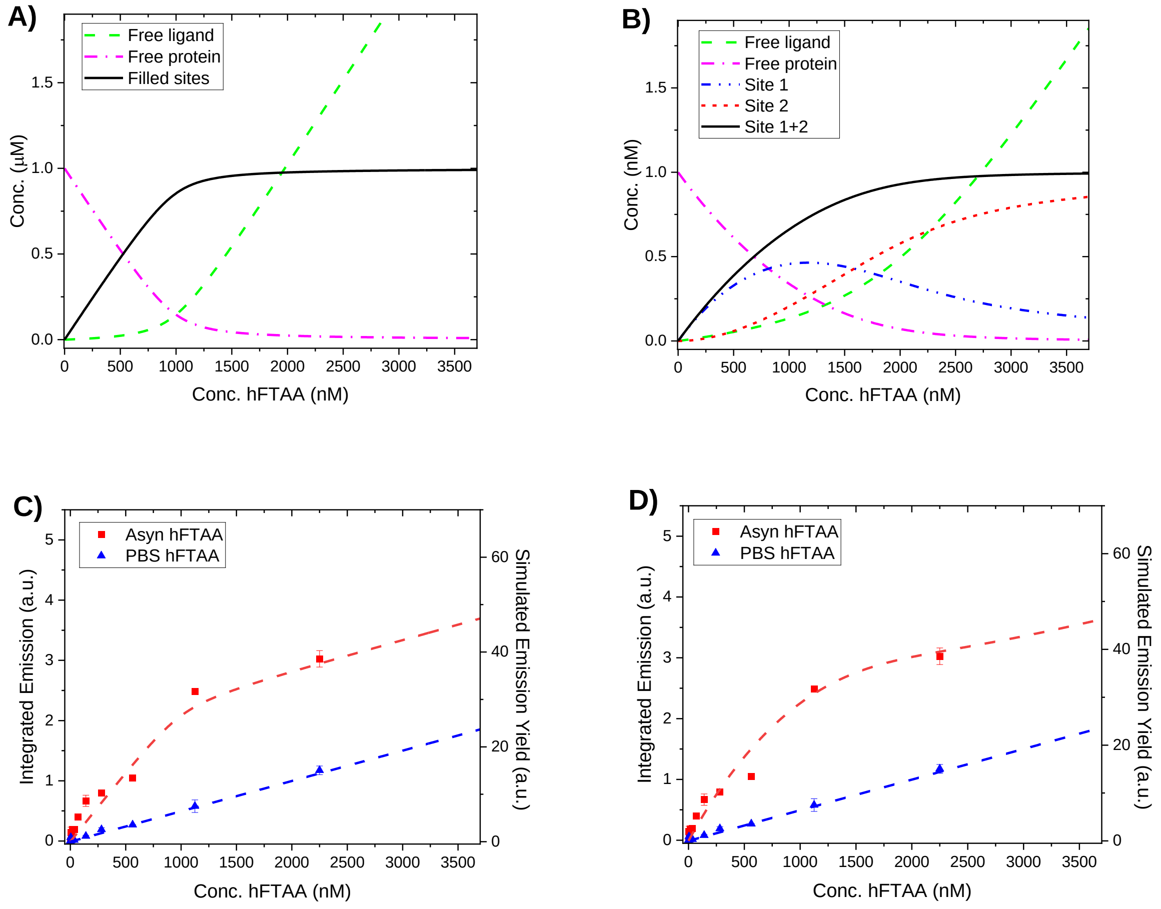

Figure S4. Binding curve plots and simulations thereof. A) Simulated populations of free ligand, free protein and bound protein in a 1000 nM solution of asyn PFF based on a 1-site model. Dissociation constant K_d = 25 nM. B) Simulated populations of free ligand, free protein and bound protein in a 1000 nM solution of asyn PFF based on a 2-site model [31]. Dissociation constants: K_{d1} = 100 nM; . K_{d1} = 300 nM. C) simulated binding curve assuming QY(h-FTAA/PFF) 30%; QY(h-FTAA/PBS) 6.4%. D) simulated binding curve assuming QY(h-FTAA/PFF-site1) 40%; QY(h-FTAA/PFF-site2) 20%; QY(h-FTAA/PBS) 6.4%.

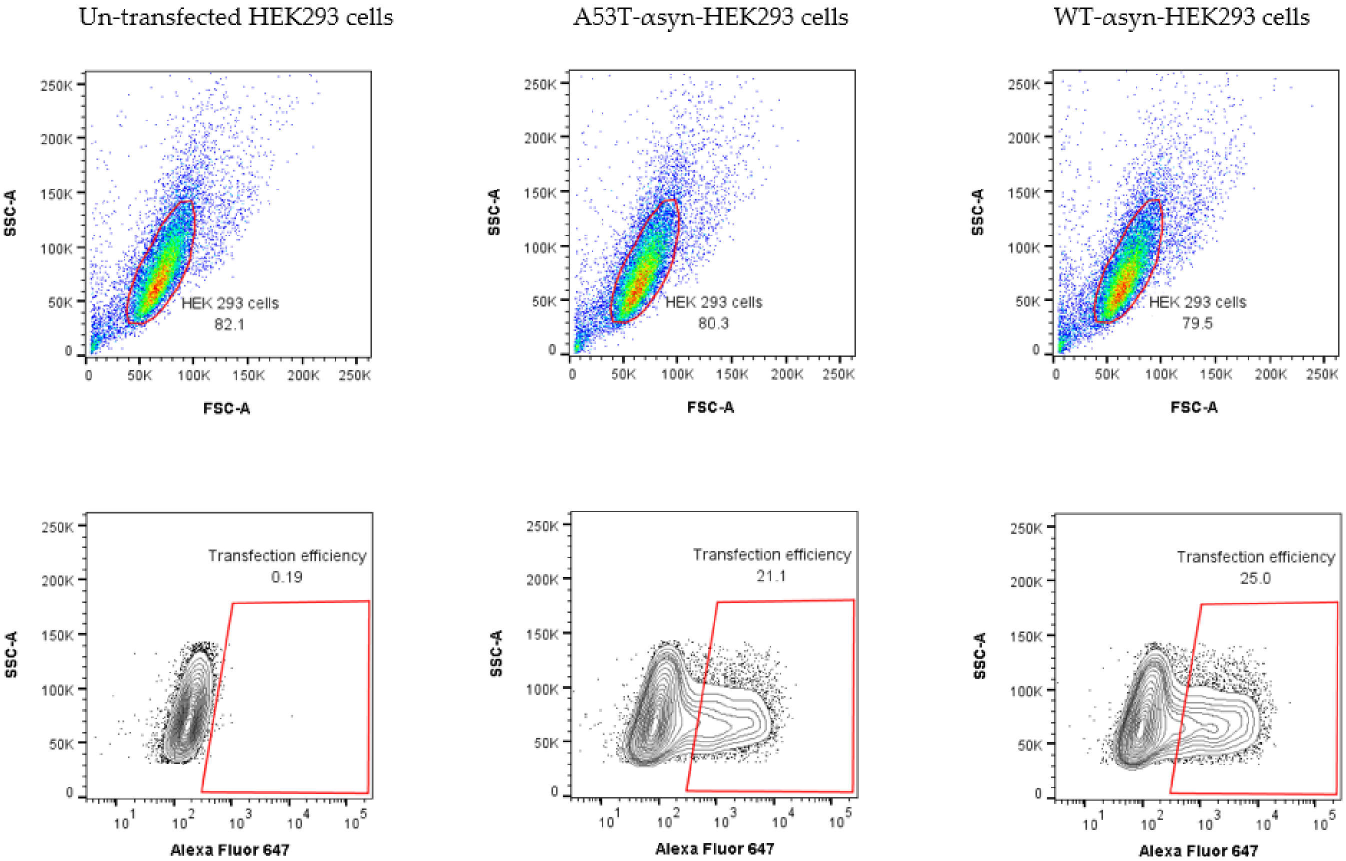

Figure S5: FACS analysis showing the efficiency of αsyn transfection in HEK293 cells expressing A53T-αsyn or WT-αsyn. The samples were excited at 633 nm. The upper panel shows the gating strategy used for selecting the main population of HEK293 cells in the forward scattering (FSC)-side scattering (SSC) plot. The un-transfected HEK293 cells were used to set the gate for both WT-αsyn and A53T-αsyn HEK293 cells. The gated population was then used for plotting the histogram, shown in lower panel as contour plots, which depicts the fraction of cells that show fluorescence from Alexa Flour 647 (illustrated in the last two plots of the lower panel).

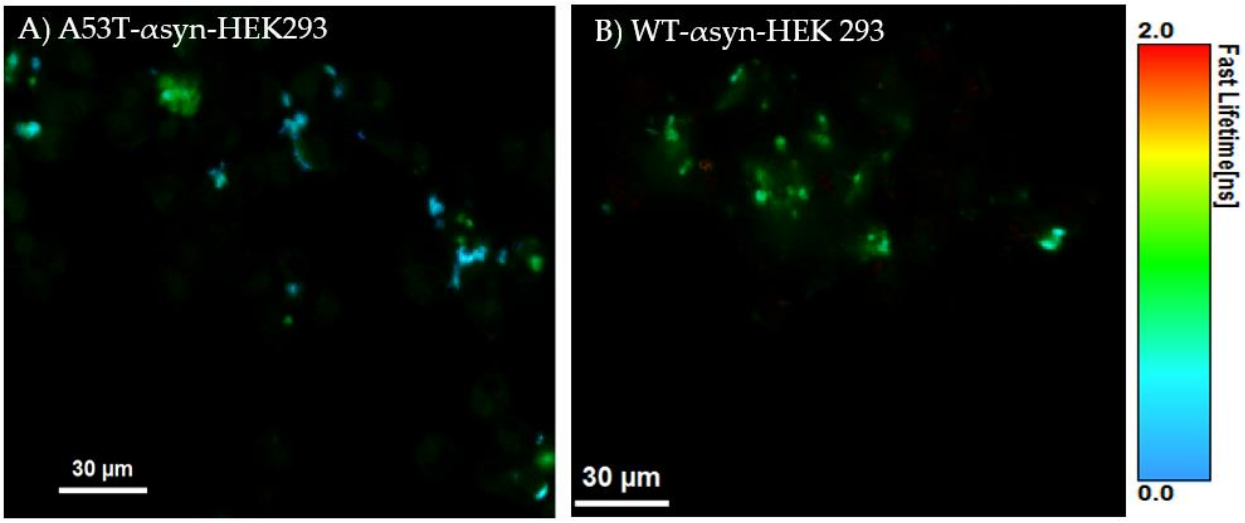

Figure S6: Representative FLIM images showing h-FTAA stained αsyn aggregates in **A)** A53T-αsyn-HEK and **B)** WT-αsyn-HEK cells. The samples were excited at 475 nm. The color bars represent the lifetime ranging from 0 ns to 2 ns.

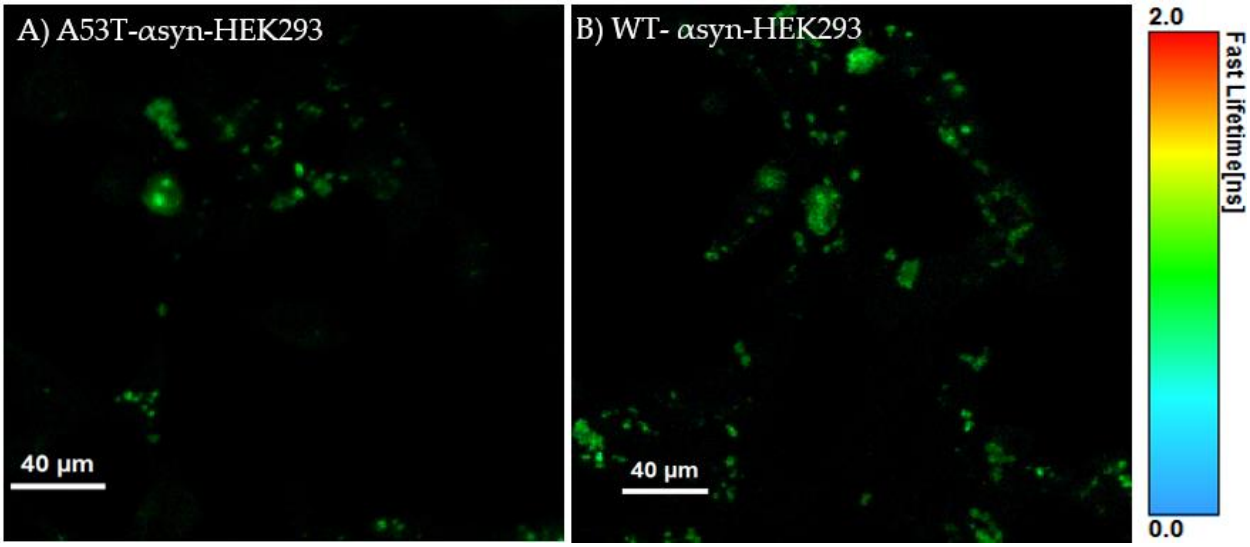

Figure S7: Representative FLIM images showing h-FTAA stained, S129P-probed αsyn aggregates in **A)** A53T-αsyn-HEK and **B)** WT-αsyn-HEK cells. The samples were excited at 475 nm. The color bars represent the lifetime ranging from 0 ns to 2 ns.

Original Western Blot:

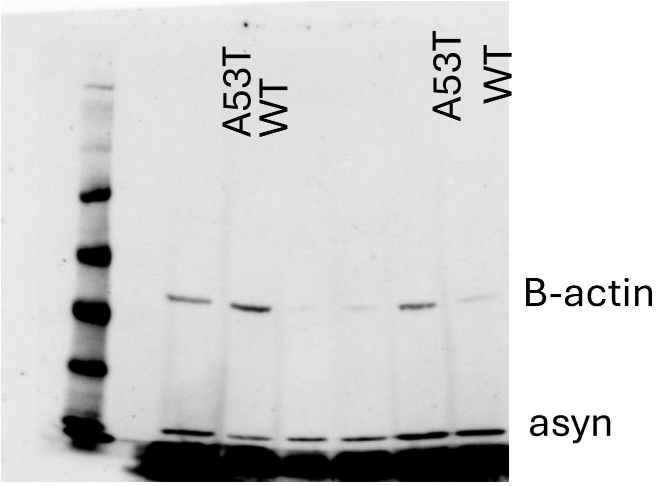

Supplement: Supplementary file 1 [file ijms-25-12458-s001.zip › ijms-3306182-Supplementary_Information_with_Westernblot.pdf]
